# Plant Proteases: From Key Enzymes in Germination to Allies for Fighting Human Gluten-Related Disorders

**DOI:** 10.3389/fpls.2019.00721

**Published:** 2019-05-29

**Authors:** Manuel Martinez, Sara Gómez-Cabellos, María José Giménez, Francisco Barro, Isabel Diaz, Mercedes Diaz-Mendoza

**Affiliations:** ^1^Centro de Biotecnologia y Genomica de Plantas, Instituto Nacional de Investigacion y Tecnologia Agraria y Alimentaria (INIA), Universidad Politécnica de Madrid (UPM), Campus Montegancedo UPM, Madrid, Spain; ^2^Departamento de Biotecnologia-Biologia Vegetal, Escuela Tecnica Superior de Ingenieria Agronomica, Alimentaria y de Biosistemas, Universidad Politécnica de Madrid (UPM), Madrid, Spain; ^3^Departamento de Mejora Genética Vegetal, Instituto de Agricultura Sostenible (IAS-CSIC), Córdoba, Spain

**Keywords:** cysteine protease, germination, proteolysis, gluten, celiac disorders

## Abstract

Plant proteases play a crucial role in many different biological processes along the plant life cycle. One of the most determinant stages in which proteases are key protagonists is the plant germination through the hydrolysis and mobilization of other proteins accumulated in seeds and cereal grains. The most represented proteases in charge of this are the cysteine proteases group, including the C1A family known as papain-like and the C13 family also called legumains. In cereal species such as wheat, oat or rye, gluten is a very complex mixture of grain storage proteins, which may affect the health of sensitive consumers like celiac patients. Since gluten proteins are suitable targets for plant proteases, the knowledge of the proteases involved in storage protein mobilization could be employed to manipulate the amount of gluten in the grain. Some proteases have been previously found to exhibit promising properties for their application in the degradation of known toxic peptides from gluten. To explore the variability in gluten-degrading capacities, we have now analyzed the degradation of gluten from different wheat cultivars using several cysteine proteases from barley. The wide variability showed highlights the possibility to select the protease with the highest potential to alter grain composition reducing the gluten content. Consequently, new avenues could be explored combining genetic manipulation of proteolytic processes with silencing techniques to be used as biotechnological tools against gluten-related disorders.

## Introduction

Plant proteases have been described to accomplish multiple roles in different physiological processes along the plant life cycle, such as programmed cell death, senescence, abscission, fruit ripening, plant growth, and N homeostasis (Grudkowska and Zagdańska, 2004; van der Hoorn, 2008; Liu et al., 2018; Tornkvist et al., 2019). In response to abiotic and biotic stresses, proteases are also involved in nutrient remobilization associated with leaf and root proteins degradation to ensure yield (Diaz-Mendoza et al., 2014; Velasco-Arroyo et al., 2016, 2018; Gomez-Sanchez et al., 2019; James et al., 2019), or in triggering the response of the plant to pathogens and phytophagous insects and acari (van der Hoorn and Jones, 2004; Shindo and van der Hoorn, 2008; Misas-Villamil et al., 2016; Diaz-Mendoza et al., 2017). Moreover, plant proteases play a crucial role in the plant seed germination, through the mobilization of other proteins accumulated in seeds and cereal grains (Grudkowska and Zagdańska, 2004; Cambra et al., 2012; Diaz-Mendoza et al., 2016; Szewinska et al., 2016; Liu et al., 2018; Radchuk et al., 2018).

Cereals grains include proteins with many different functions. Around 80% of these proteins are storage proteins, packed in the endosperm together with starch and lipids (Shewry et al., 1995; Shewry and Halford, 2002). These proteins are synthesized during grain development and maturation, subsequently included as storage proteins, and finally degraded during germination. Several groups of proteases have been implicated in seed germination. The most represented proteases in charge of the mobilization and degradation of storage proteins are the cysteine proteases (CysProt) (Grudkowska and Zagdańska, 2004; Tan-Wilson and Wilson, 2011; Szewinska et al., 2016). Among CysProt, the C1A family known as papain-like and the C13 family also called legumains or vacuolar processing enzymes (VPEs), have been the most studied (Hara-Nishimura et al., 1995, 1998; Kinoshita et al., 1999; Grudkowska and Zagdańska, 2004; Prabucka and Bielawski, 2004; Martínez and Díaz, 2008; Shi and Xu, 2009; Szewinska et al., 2016).

Pharmaceutical, food and beverage, detergent, and biofuel industries have for long time exploited enzyme catalysis in commercial-scale applications, being the use of papain an example of success in food industry over the last four decades (Fernández-Lucas et al., 2017). In recent years, newly identified plant proteases have been found to exhibit promising properties for their application in the food industry (Feijoo-Siota and Villa, 2011). A case of particular interest is the degradation of peptides toxic for celiac patients by proteases involved in the germination of cereals (Hartmann et al., 2006; Kiyosaki et al., 2007; Stenman et al., 2009, 2010). In particular, the 33-mer peptide of gluten is the most immunogenic peptide known so far. It is resistant to human proteases and responsible for eliciting about 90% of the allergic response induced by the full complement of wheat proteins (Tye-Din et al., 2010). The immunogenic peptide can be hydrolyzed by a combination of at least six different peptidases from sourdough lactobacilli and fungal proteases provided as a food supplement (De Angelis et al., 2010). However, the finding and use of plant proteases have several advantages compared to bacterial and fungal peptidases; they are natural components of harmless food and, unlike the fungal proteases, their safety must not be proven with potential benefits for both therapeutics and food processing (Comino et al., 2013). Therefore, the co-administration of exogenous proteases with food is a very appealing therapeutic treatment for celiac patients by facilitating gluten degradation. In particular, the knowledge of the proteases involved in storage protein mobilization could be employed to discover proteases able to degrade gluten efficiently. Several pathologies have been described with gluten intake: celiac disease (CD), an autoimmune disorder with a prevalence of about 0.7–2% in the human population (Rewers, 2005) that has increased in the last fifty years (Rubio-Tapia et al., 2009); and non-celiac wheat sensitivity (NCWS), a new pathology which is estimated to occur in about 6% of the population in western countries (Sapone et al., 2011).

## Plant Proteases in the Germination Process of Barley

In cereal grains, different proteases have been involved in the germination process (Müntz et al., 2002; Grudkowska and Zagdańska, 2004; Tan-Wilson and Wilson, 2011; Szewinska et al., 2016). In maize, wheat and barley the CysProts are responsible of around 90% of the proteolytic activity (De Barros and Larkins, 1994; Zhang and Jones, 1995; Bottari et al., 1996). Plant CysProts from the papain family (C1A) (Mikkonen et al., 1996; Prabucka and Bielawski, 2004; Shi and Xu, 2009; Cambra et al., 2012; Diaz-Mendoza et al., 2016) and CysProts of the legumain family (C13) (Hara-Nishimura et al., 1998; Radchuk et al., 2011, 2018), are the most representative groups in proteolysis and mobilization of storage proteins in cereal grain. Other proteases have been implicated in the germination process in cereal grains, such as the S10 serine carboxypeptidases (Ser-) (Dal Degan et al., 1994; Washio and Ishikawa, 1994; Dominguez and Cejudo, 1999; Domínguez et al., 2002; Li et al., 2016).

**FIGURE 1:**
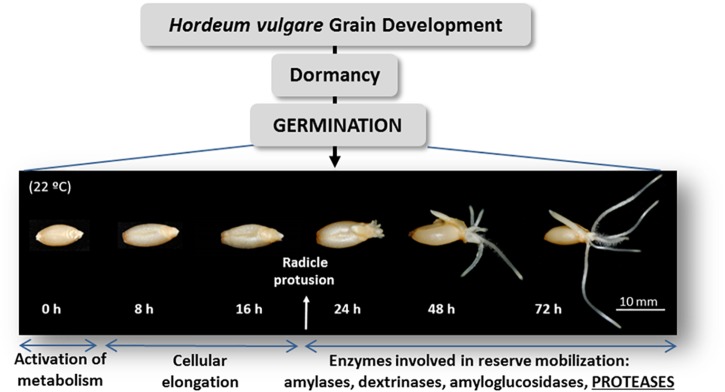
Schematic representation of *Hordeum vulgare* grain germination.

Barley is one of the grain cereals further studied. Proteases, amylases, dextrinases and other hydrolases are crucial for the survival of the seedling until the photosynthesis is finally established (Figure 1). In the barley germination, proteases of the papain-like C1A (Koehler and Ho, 1988, 1990a,b; Poulle and Jones, 1988; Zhang and Jones, 1995; Sreenivasulu et al., 2008) and the legumain-like C13 families (Linnestad et al., 1998; Radchuk et al., 2011, 2018) have mainly been identified. Besides, the expression of six serine carboxypeptidases (Ser-CPs) during maturation and germination of the barley grain has been documented (Dal Degan et al., 1994; Table 1).

**Table 1:** Example of *Hordeum vulgare* proteases involved in germination.

Protease family	*Hordeum vulgare* proteases	References
C1A	Cathepsin L-like (EP-A and EP-B) (HvPap-4, -6 and -16)	Koehler and Ho, 1988, 1990a,b; Poulle and Jones, 1988; Mikkonen et al., 1996; Martínez et al., 2009
	Cathepsin H-like (aleurain)	Rogers et al., 1985; Holwerda and Rogers, 1992
	Cathepsin B-like (HvCathB)	Martínez et al., 2003
	Cathepsin F-like (HvPap-1)	Sreenivasulu et al., 2008; Cambra et al., 2012; Diaz-Mendoza et al., 2016
C13	Legumains-VPEs (HvLeg-2, -3, -7)	Linnestad et al., 1998; Radchuk et al., 2011; Julián et al., 2013
	Legumains-VPEs (HvLeg-5 also called HvVPE4)	Radchuk et al., 2011, 2018
Ser-CP	Six Serine Carboxypeptidases (Ser-CPs)	Dal Degan et al., 1994

Two cathepsin L-like, EP-A and EP-B, were the first barley CysProts described to participate in the proteolytic degradation of the storage proteins in barley grains (Koehler and Ho, 1988, 1990a,b; Poulle and Jones, 1988; Mikkonen et al., 1996). These CysProts are synthesized in the scutellar epithelium and the aleurone layer and, then, secreted to the endosperm upon germination (Mikkonen et al., 1996; Martínez et al., 2009). An aleurain belonging to the cathepsin H-like group of CysProts has been isolated from aleurone (Rogers et al., 1985; Holwerda and Rogers, 1992) and a cathepsin B-like protein, HvCathB, has been detected in aleurone and developing endosperm (Martínez et al., 2003). Similarly, a member of the cathepsin F-like group, the HvPap-1, has been detected in germinating barley grains (Sreenivasulu et al., 2008). HvPap-1 takes part in the proteolytic mobilization of stored proteins like hordeins, albumins and globulins in the barley endosperm and its activity is modulated by its own propeptide (Cambra et al., 2012). HvPap-1 contributes to barley grain filling and germination since over-expression or silencing of *HvPap-1* gene in barley transgenic plants alters the metabolite composition of the grain, and modifies the germination process by delaying or accelerating it (Diaz-Mendoza et al., 2016). Likewise, the presence of HvPap-4, -6 and -10 in the germinating embryo and aleurone layers has been demonstrated, as well as the capacity of hordein degradation by HvPap-6 and 10 (Martínez et al., 2009). Regarding the barley C13 legumain-like family, their ability to process other CysProts in order to activate them to take part in the proteolytic degradation of the storage proteins it has been also described (Cambra et al., 2010). This is the case of the HvLeg-2 legumain of barley, which is highly expressed during germination and could be involved in the mobilization of storage proteins either by direct proteolytic degradation or by processing and activation of other CysProts (Cambra et al., 2010; Julián et al., 2013). Consequently, for the hydrolysis and mobilization of storage proteins in barley grains the cooperative proteolysis of papain-like CysProt and legumains is essential (Cambra et al., 2010). Overall, the diversity and proteolytic specificity of barley proteases involved in the degradation of storage compounds makes them a starting point to select a potential candidate for further applications.

## Potential Application of Barley Proteases in Celiac Disease

From an applied point of view, this wide knowledge on barley proteases acting in the germination process could be employed to manipulate and improve the grain composition not only in barley but also in those cereal species such as wheat, oat, rye and related species and hybrids, in which a number of proteins, known as gluten proteins, affect the health of sensitive consumers.

Celiac disease is the best-characterized pathology associated with gluten consumption and there is a major environmental factor, the ingestion of gluten proteins not only from wheat but also from barley and rye. It has also an important genetic risk factor related to the genes encoding for the human leukocyte antigen (HLA) -DQ2 or -DQ8 (Schuppan, 2000). A lifelong gluten free diet reverses signs and symptoms of celiac disease and NCWS. Nevertheless, this is difficult to follow due to the wide gluten presence in many diet foods. Gluten is a very complex mixture of storage proteins classified into glutenins and gliadins, comprising around 80% of the total grain proteins, with about 30% gliadins and 50% glutenins. Most of the CD related epitopes have been found in the gliadin fraction (Arentz-Hansen et al., 2002). Gliadins are rich in the amino acids proline, and glutamine, which make them resistant to being fully digested in the gastrointestinal tract. This is the case of human proteases, which do not accept proline at their cleavage sites. Partial digestion of gluten generates small peptides which induces a CD4+ T-cell inflammatory response leading to villous atrophy through a two-signal model (Brandtzaeg, 2006). According to this model, a first innate immune response is triggered by certain peptides, such as the 19-mer gliadin peptide, resulting in the production of interleukin 15 (IL-15) by epithelial cells. The result is the disruption of the epithelial barrier by increasing its permeability. Then, other immune-adaptive peptides, like the 33-mer, can reach the lamina propria and they are deaminated by the tissue transglutaminase (tTG2), providing a strong negative charge to gliadin peptide enhancing their affinity to bind within the HLA-DQ2/8 bound (Bethune and Khosla, 2012).

Different therapeutic alternatives are being developed, as the inhibition of transglutaminase, the antagonism of peptide binding to HLA-DQ2 or HLA-DQ8, the enzymatic detoxification of gluten, or the introduction of natural amino acid substitution to eliminate toxicity (Schuppan et al., 2009; Ruiz-Carnicer et al., 2019). Some other strategies have been developed trying to relieve the negative effect of gluten proteins (Scherf et al., 2018). A promising approach is the down-regulation of genes encoding for gliadins by RNAi technologies, generating transgenic wheat lines with low levels of toxicity for celiacs (Gil-Humanes et al., 2010). Previous investigations have already developed wheat with a down regulation of gliadins expression by hairpin technology. As result, a very low or null T-cells stimulation was proven (Gil-Humanes et al., 2010). The reduced-gliadin breads showed baking and sensory properties, and overall acceptance, similar to those of normal flour, but with up to 97% lower gliadin content (Gil-Humanes et al., 2014). Their results showed that targeting of genes related to celiac disease is feasible and may reduce T-cell epitopes. More recent, low-gluten wheat was engineered with CRISPR/Cas9 technology (Jouanin et al., 2018; Sánchez-León et al., 2018), providing bread and durum with reduced amount of α-gliadins in the seed kernel and a low immunoreactivity for gluten intolerant consumers.

In addition, enzymes able to degrade gliadins have been suggested as hopeful therapeutic agents, as is the barley CysProt EP-B2, which efficiently hydrolyzed a recombinant wheat gluten protein, α2-gliadin, which contains sequences with known immunotoxicity in celiac sprue patients (Bethune et al., 2006). Some prolyl endoproteases from *Aspergillus niger* (AN-PEP) and *Sphingomonas capsulate* (SC-PEP), with also gliadin degradative capacity, have been characterized (Gass et al., 2007). An EP-B2 and SC-PEP combination has been studied as a promising therapeutic tool, being EP-B2 responsible for hydrolyzing gluten proteins into short oligopeptides, which are still toxic, and SC-PEP for breaking down these oligopeptides into non-toxic metabolites (Gass et al., 2007).

## Wheat Gluten Hydrolysis by Barley Cysprot

To further explore the enzymatic strategy, we have now analyzed the degradation of six different gliadin fractions from wheat cultivars and breeding lines (Perico, THA1, THA7, THA53, BW2003, and BW208) (Gil-Humanes et al., 2012) by four different CysProt from barley (HvPap-1, -4, -6, and -16) (Supplementary Material). These proteases have been previously identified, characterized and purified in *Escherichia coli* (Martínez et al., 2009). With the use of these four CysProt as a representation of different groups of L-like cathepsins and F-like cathepsins, a wide view of their activity on gliadin proteolysis has been achieved. The final goal has been to explore their potential to reduce gliadin content in the wheat gluten or to use them in therapy treatments.

**FIGURE 2:**
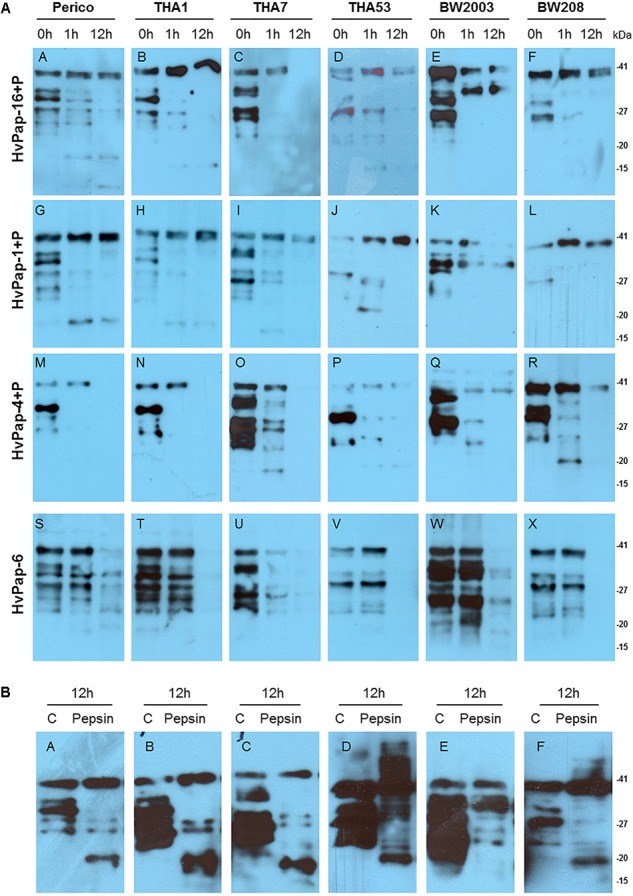
**(A)** Western blots of 8 μg of gliadins from Perico, THA1, THA7, THA53, BW2003, and BW208 wheat cultivars, incubated with 2 μg of each barley CysProt for 0, 1, and 12 h at 28 °C. Gliadin incubation with HvPap-16+P **(A–F)**; HvPap-1+P **(G–L)**; HvPap-4+P **(M–R)**; HvPap-6 **(S–X)**. **(B)** Gliadins stability assays after 12 h of incubation without pepsin as control and gliadin treatments with 6 μg of pepsin for 12 h **(A–F)**. Immunoblotting was performed with Gluten Tox G12 HRP-conjugate antibody, 200× (Biomedal diagnostics) following the manufacturer instructions. Pepsin (P) and molecular weight in kilo Dalton unit (kDa).

Hydrolysis of the different wheat gliadins by purified barley CysProt was observed by western blot analysis. When gliadins from the six wheat cultivars were treated with HvPap-1, -4, -6, and -16 for 1 and 12 h, different degradation patterns by each CysProt were observed (Figure 2A). Degradation in all gliadin samples was appreciated after 1 h of incubation with HvPap-1, -4, -6, and -16 proteases. In most cases, higher bands remained stable while lower molecular weight bands started to disappear after 1 h of treatment. After 12 h of incubation with barley proteases, an increased differential degradation of gliadin bands was observed. Whereas higher molecular weight bands remained stable after HvPap-16 and HvPap-1 treatments (Figure 2A,A–L), degradation was almost complete after HvPap-4 and HvPap-6 action (Figure 2A,M–X). To discard any processing or degradation effect due to instability or autohydrolysis, gliadins were incubated for 12 h without proteases (Figure 2B). Likewise, as HvPap-1, -4 and -16 were activated by adding pepsin, an analysis of gliadins stability in presence of this commercial peptidase was carried out (Figure 2B). Although some smaller bands were hydrolysed by pepsin, in all treatments the band with higher molecular weight remains stable after 12 h incubation, in contrast with the total degradation exerted by HvPap-4 and HvPap-6. These proteases seem to be the most promising gliadin-degrading enzymes. Furthermore, as HvPap-6 does not need to be activated by pepsin, this protease should be selected over HvPap-4 since degradation is only due to its own activity.

## Plant Proteases and Celiac Disease: Future Perspectives

The potential of plant proteases to alter grain content and the mobilization of stored proteins greatly supports their use as biotechnological tools. As an example, gluten proteins responsible of celiac disease and other gluten-related pathologies are suitable targets for these enzymes. For gluten intolerant patients, the only available treatment is a gluten-free diet, which is extremely difficult to maintain due to gluten ubiquity in human diets. Given the negative impact of quality of life of celiac patients, there is an urgent need to develop food suitable for them. In our research, we have provided new information on the potential of plant CysProt to reduce gliadin content. HvPap-6 CysProt ranks as the best candidate given the encouraging results of this study. The final aim would be the development of transgenic lines of wheat overexpressing this CysProt under endosperm specific promoters to reduce gluten toxicity. This cereal would be used afterward to obtain flour with which to elaborate products suitable for humans with this autoimmune enteropathy. Transgenic wheat lines overexpressing the *HvPap-6* gene from barley would have advantages in comparison of oral enzyme therapy since consumers will not be exposed to harmful gluten. Unwanted reactions in celiac patients probably would decrease by consuming lower or even null levels of toxic gluten from transgenic wheat lines. Administration of hydrolytic glutenases as food supplement is an alternative to deliver the therapeutic agents directly to the small intestine, for further degradation of wheat gluten. One step beyond would be to produce transgenic wheat lines combining the overexpression of a CysProt with high gliadin-degrading efficiency and the downregulation of the expression of gliadins using genome editing techniques. The next step should be to evaluate the *in vivo* efficiency of selected proteases for degrading wheat gliadins in the endosperm. For that, overexpression of these proteases under the control of endosperm specific promoters, such as those from HMW, gamma or alpha-gliadins would be necessary. A recent publication by Osorio et al. (2019), expressing an endoprotease from barley reinforced our perspective about this approach. These authors overexpressed the barley protease EP-B2, combined with prolyl endopeptidases from bacterium, under the control of the endosperm specific wheat *1Dy* high-molecular wheat glutenin promoter (pHMWg). A significant reduction (up to 67%) of the amount of the indigestible gluten peptides of all prolamin families was achieved. This approach will contribute, in combination with other strategies like CRISPR/Cas9, to provide wheat varieties suitable for celiac and other gluten intolerance patients. Overall, with new goals, the potential of exploring the properties of plant proteases acting along seed germination will open a great field of research, as a biotechnological alternative to provide new prevention strategies in protein-caused disorders. In conclusion, this is an alternative approach for enzymatic gluten degradation to generate gluten-free wheat to manufacture gluten free products.

## Data Availability

All datasets generated for this study are included in the manuscript and/or the Supplementary Files.

## Author Contributions

ID, FB, MM, and MD-M conceived the research. SG-C, MG, and MD-M performed the experimental research. All authors contributed to the final version of the manuscript, and read and approved the final manuscript.

## Conflict of Interest Statement

The authors declare that the research was conducted in the absence of any commercial or financial relationships that could be construed as a potential conflict of interest.

## References

[B1] Arentz-Hansen H. Mc Adam S. N. Molberg Ø Fleckenstein B. Lundin K. E. Jørgensen T. J. (2002). Celiac lesion T cells recognize epitopes that cluster in regions of gliadins rich in proline residues. *Gastroenterology* 123 803–809. 10.1053/gast.2002.35381 12198706

[B2] Bethune M. T. Khosla C. (2012). Oral enzyme therapy for celiac sprue. *Methods Enzymol.* 502 241–271. 10.1016/B978-0-12-416039-2.00013-6 22208988 PMC3382113

[B3] Bethune M. T. Strop P. Tang Y. Sollid L. M. Khosla C. (2006). Heterologous expression, purification, refolding and structural-functional characterization of EP-B2, a self-activating barley cysteine endoprotease. *Chem. Biol.* 13 637–647. 10.1016/j.chembiol.2006.04.008 16793521

[B4] Bottari A. Capocchi A. Galleschi A. Jopova A. Saviozzi E. (1996). Asparaginyl endopeptidase during maturation and germination of durum wheat. *Physiol. Plant.* 97 475–480. 10.1111/j.1399-3054.1996.tb00506.x

[B5] Brandtzaeg P. (2006). The changing immunological paradigm in coeliac disease. *Immunol. Lett.* 105 127–139. 10.1016/j.imlet.2006.03.004 16647763

[B6] Cambra I. Garcia F. J. Martínez M. (2010). Clan CD of cysteine peptidases as an example of evolutionary divergences in related protein families across plant clades. *Gene* 449 59–69. 10.1016/j.gene.2009.09.003 19751811

[B7] Cambra I. Martínez M. Dáder B. González-Melendi P. Gandullo J. Santamaría M. E. (2012). A cathepsin F-like peptidase involved in barley grain protein mobilization, HvPap-1, is modulated by its own propeptide and by cystatins. *J. Exp. Bot.* 63 4615–4629. 10.1093/jxb/ers137 22791822 PMC3421991

[B8] Comino I. Moreno M. de L. Real A. Rodriguez-Herrera A. Barro F. (2013). The gluten-free diet: testing alternative cereals tolerated by celiac patients. *Nutrients* 5 4250–4268. 10.3390/nu5104250 24152755 PMC3820072

[B9] Dal Degan F. Rocher A. Cameron-Mills V. von Wettstein D. (1994). The expression of serine carboxypeptidases during maturation and germination of the barley grain. *Proc. Natl. Acad. Sci. U.S.A.* 91 8209–8213. 10.1073/pnas.91.17.8209 7520177 PMC44575

[B10] De Angelis M. Cassone A. Rizzello C. G. Gagliardi F. Minervini F. Calasso M. (2010). Mechanism of degradation of immunogenic gluten epitopes from *Triticum turgidum* L. var. durum by sourdough lactobacilli and fungal proteases. *Appl. Environ. Microbiol.* 76 508–518. 10.1128/AEM.01630-09 19948868 PMC2805216

[B11] De Barros E. G. Larkins B. A. (1994). Cloning of a cDNA encoding a putative cysteine protease gliadin during wheat germination. *Phytochemistry* 43Z, 39–44. 10.1016/0168-9452(94)90176-7

[B12] Diaz-Mendoza M. Dominguez-Figueroa J. D. Velasco-Arroyo B. Cambra I. Gonzalez-Melendi P. Lopez-Gonzalvez A. (2016). HvPap-1 C1A protease and HvCPI-2 cystatin contribute to barley grain filling and germination. *Plant Physiol.* 170 2511–2524. 10.1104/pp.15.01944 26912343 PMC4824613

[B13] Diaz-Mendoza M. Velasco-Arroyo B. Gonzalez-Melendi P. Martinez M. Diaz I. (2014). C1A cysprot-cystatin interactions in leaf senescence. *J. Exp. Bot.* 65 3825–3833. 10.1093/jxb/eru043 24600023

[B14] Diaz-Mendoza M. Velasco-Arroyo B. Santamaria M. E. Diaz I. Martinez M. (2017). HvPap-1 C1A protease participates differentially in the barley response to a pathogen and an herbivore. *Front. Plant Sci.* 12:1585. 10.3389/fpls.2017.01585 28955371 PMC5601043

[B15] Dominguez F. Cejudo F. J. (1999). Patterns of starchy endosperm acidification and protease gene expression in wheat grains following germination. *Plant Physiol.* 119 81–88. 10.1104/pp.119.1.81 9880349 PMC32245

[B16] Domínguez F. González M. C. Cejudo F. J. (2002). A germination-related gene encoding a serine carboxypeptidase is expressed during the differentiation of the vascular tissue in wheat grains and seedlings. *Planta* 215 727–734. 10.1007/s00425-002-0809-2 12244437

[B17] Feijoo-Siota L. Villa T. G. (2011). Native and biotechnologically engineered plant proteases with industrial applications. *Food Biopro. Technol.* 4 1066–1088. 10.1007/s11947-010-0431-4

[B18] Fernández-Lucas J. Castañeda D. Hormigo D. (2017). New trends for a classical enzyme: papain, a biotechnological success story in the food industry. *Trends Food Sci. Technol.* 68 91–101. 10.1016/j.tifs.2017.08.017

[B19] Gass J. Bethune M. T. Siegel M. Spencer A. Khosla C. (2007). Combination enzyme therapy for gastric digestion of dietary gluten in patients with celiac sprue. *Gastroenterology* 133 472–480. 10.1053/j.gastro.2007.05.028 17681168

[B20] Gil-Humanes J. Pistón F. Altamirano-Fortoul R. Real A. Comino I. Sousa C. (2014). Reduced-gliadin wheat bread: an alternative to the gluten-free diet for consumers suffering gluten-related pathologies. *PLoS One* 9:e90898. 10.1371/journal.pone.0090898 24621595 PMC3951262

[B21] Gil-Humanes J. Pistón F. Giménez M. J. Martín A. Barro F. (2012). The intogression of RNAi silencing of γ-gliadins into commercial lines of bread wheat changes the mixing and technological properties of the dough. *PLoS One* 7:e45937. 10.1371/journal.pone.0045937 23029328 PMC3454332

[B22] Gil-Humanes J. Pistón F. Tollefsen S. Sollid L. M. Barro F. (2010). Effective shutdown in the expression of celiac disease-related wheat gliadin T-cell epitopes by RNA interference. *Proc. Natl. Acad. Sci. U.S.A.* 107 17023–17028. 10.1073/pnas.1007773107 20829492 PMC2947919

[B23] Gomez-Sanchez A. Gonzalez-Melendi P. Santamaria M. E. Arbona V. Lopez-Gonzalvez A. Garcia A. (2019). Repression of drought-induced cysteine-protease genes alter barley leaf structure and the response to abiotic and biotic stresses. *J. Exp. Bot.* 70 2143–2155. 10.1093/jxb/ery410 30452688

[B24] Grudkowska M. Zagdańska B. (2004). Multifunctional role of plant cysteine proteinases. *Acta Biochim. Pol.* 51 609–624. 15448724

[B25] Hara-Nishimura I. Kinoshita T. Hiraiwa N. Nishimura M. (1998). Vacuolar processing enzymes in protein-storage vacuoles and lytic vacuoles. *J. Plant Physiol.* 152 668–674. 10.1016/S0176-1617(98)80028-X

[B26] Hara-Nishimura I. Shimada T. Hiraiwa N. Nishimura M. (1995). Vacuolar processing enzyme responsible for maturation of seed protein. *J. Plant Physiol.* 145 6412–6417. 10.1016/S0176-1617(11)81275-7

[B27] Hartmann G. Koehler P. Wieser H. (2006). Rapid degradation of gliadin peptides toxic for celiac disease patients by proteases from germinating cereals. *J. Cereal Sci.* 44 368–371. 10.1016/j.jcs.2006.10.002

[B28] Holwerda B. C. Rogers J. C. (1992). Purification and characterization of aleurain: a plant thiol protease functionally homologous to mammalian cathepsin h. *Plant Physiol.* 99 848–855. 10.1104/pp.99.3.848 16669011 PMC1080555

[B29] James M. Masclaux-Daubresse C. Marmagne A. Azzopardi M. Laîné P. Goux D. (2019). A new role for SAG12 cysteine protease in roots of *Arabidopsis thaliana*. *Front. Plant Sci.* 9:1998. 10.3389/fpls.2018.01998 30687379 PMC6337903

[B30] Jouanin A. Boyd L. Visser R. G. F. Smulders M. J. M. (2018). Development of wheat with hypoimmunogenic gluten obstructed by the gene editing policy in Europe. *Front. Plant Sci.* 9:1523. 10.3389/fpls.2018.01523 30405661 PMC6200864

[B31] Julián I. Gandullo J. Santos-Silva L. K. Diaz I. Martínez M. (2013). Phylogenetically distant barley legumains have a role in both seed and vegetative tissues. *J. Exp. Bot.* 64 2929–2941. 10.1093/jxb/ert132 23669572

[B32] Kinoshita T. Yamada K. Hiraiwa N. Nishimura M. Hara-Nishimura I. (1999). Vacuolar processing enzyme is up-regulated in the lytic vacuoles of vegetative tissues during senescence and under various stressed conditions. *Plant J.* 19 43–53. 10.1046/j.1365-313X.1999.00497.x 10417725

[B33] Kiyosaki T. Matsumoto I. Asakura T. Funaki J. Kuroda M. Misaka T. (2007). Gliadain, a gibberellin-inducible cysteine proteinase occurring in germinating seeds of wheat, *Triticum aestivum* L., specifically digests gliadin and is regulated by intrinsic cystatins. *FEBS J.* 164 470–477. 10.1111/j.1742-4658.2007.05749.x 17371549

[B34] Koehler S. M. Ho T. D. (1988). Purification and characterization of gibberelic acid-induced cysteine endoproteases in barley aleurone layers. *Plant Physiol.* 87 251–258. 10.1104/pp.87.1.95 16666134 PMC1054705

[B35] Koehler S. M. Ho T. D. (1990a). A major gibberellic acid-induced barley aleurone cysteine proteinase which digests hordein. *Plant Physiol.* 94 251–258. 10.1104/pp.94.1.251 16667694 PMC1077218

[B36] Koehler S. M. Ho T. D. (1990b). Hormonal regulation, processing, and secretion of cysteine proteinases in barley aleurone layers. *Plant Cell* 2 769–783. 10.1105/tpc.2.8.769 2152126 PMC159929

[B37] Li Z. Tang L. Qiu J. Zhang W. Wang Y. Tong X. (2016). Serine carboxypeptidase 46 regulates grain filling and seed germination in rice (*Oryza sativa* L.). *PLoS One* 11:e0159737. 10.1371/journal.pone.0159737 27448032 PMC4957776

[B38] Linnestad C. Doan D. N. P. Brown R. C. Lemmon B. E. Meyer D. J. Jung R. (1998). Nucellain, a barley homolog of the dicot vacuolar-processing protease is localized in nucellar cell walls. *Plant Physiol.* 118 1169–1180. 10.1104/pp.118.4.1169 9847091 PMC34733

[B39] Liu H. Hu M. Wang Q. Cheng L. Zhang Z. (2018). Role of papain-like cysteine proteases in plant development. *Front. Plant Sci.* 9:1717. 10.3389/fpls.2018.01717 30564252 PMC6288466

[B40] Martínez M. Cambra I. Carrillo L. Diaz-Mendoza M. Diaz I. (2009). Characterization of the entire cystatin gene family in barley and their target cathepsin L-like cysteine proteases, partners in the hordein mobilization during seed germination. *Plant Physiol.* 151 1531–1545. 10.1104/pp.109.146019 19759340 PMC2773090

[B41] Martínez M. Díaz I. (2008). The origin and evolution of plant cystatins and their target cysteine proteinases indicate a complex functional relationship. *BMC Evol. Biol.* 8:198. 10.1186/1471-2148-8-198 18616807 PMC2474614

[B42] Martínez M. Rubio-Somoza I. Carbonero P. Diaz I. (2003). A cathepsin B-like CysProt gene from *Hordeum vulgare* (gene catb) induced by GA in aleurone cells is under circadian control in leaves. *J. Exp. Bot.* 54 951–959. 10.1093/jxb/erg099 12598566

[B43] Mikkonen A. Porali I. Cercós M. Ho T. D. (1996). Major cysteine proteinase, EPB, in germinating barley seeds: structure of two intronless genes and regulation of expression. *Plant Mol. Biol.* 31 239–254. 10.1007/BF00021787 8756590

[B44] Misas-Villamil J. C. Van Der Hoorn R. A. Doehlemann G. (2016). Papainlike cysteine proteases as hubs in plant immunity. *New Phytol.* 212 902–907. 10.1111/nph.14117 27488095

[B45] Müntz K. Blattner F. R. Shutov A. D. (2002). Legumains – a family of asparagine-specific cysteine endopeptidases involved in propolypeptide processing and protein breakdown in plants. *J. Plant Physiol.* 160 1281–1293. 10.1078/0176-1617-00853

[B46] Osorio C. E. Wen N. Mejias J. H. Liu B. Reinbothe S. von Wettstein D. (2019). Development of wheat genotypes expressing a glutamine-specific endoprotease from barley and a prolyl endopeptidase from *Flavobacterium meningosepticum* or *Pyrococcus furiosus* as a potential remedy to celiac disease. *Funct. Integr. Genomics* 19:123. 10.1007/s10142-018-0632-x 30159724 PMC6311431

[B47] Poulle M. Jones B. L. (1988). A proteinase from germinating barley. Purification and some physical properties. *J. Plant Physiol.* 88 1454–1460. 10.1104/pp.88.4.1454 16666480 PMC1055779

[B48] Prabucka B. Bielawski W. (2004). Purification and partial characteristic of major gliadin-degrading cysteine endopeptidase from germinating triticale seeds. *Acta Physiol. Plant.* 26 383–391. 10.1007/s11738-004-0027-6

[B49] Radchuk V. Tran V. Radchuk R. Diaz-Mendoza M. Weier D. Fuchs J. (2018). Vacuolar processing enzyme 4 controls grain size in barley by executing programmed cell death in pericarp. *New Phytol.* 218 1127–1142. 10.1111/nph.14729 28836669

[B50] Radchuk V. Weier D. Radchuk R. Weschke W. Weber H. (2011). Development of maternal seed tissue in barley is mediated by regulated cell expansion and cell disintegration and coordinated with endosperm growth. *J. Exp. Bot.* 62 1217–1227. 10.1093/jxb/erq348 21059741 PMC3022404

[B51] Rewers M. (2005). Epidemiology of celiac disease: what are the prevalence, incidence, and progression of celiac disease? *Gastroenterology* 128 S47–S51. 10.1053/j.gastro.2005.02.030 15825126

[B52] Rogers J. C. Dean D. Heck G. R. (1985). Aleurain: a barley thiol protease closely related to mammalian cathepsin H. *Proc. Natl. Acad. Sci. U.S.A.* 82 6512–6516. 10.1073/pnas.82.19.6512 3901004 PMC390747

[B53] Rubio-Tapia A. Kyle R. A. Kaplan E. L. Johnson D. R. Page W. Erdtmann F. (2009). Increased prevalence and mortality in undiagnosed celiac disease. *Gastroenterlogy* 137 88–93. 10.1053/j.gastro.2009.03.059 19362553 PMC2704247

[B54] Ruiz-Carnicer Á Comino I. Segura V. Ozuna C. V. Moreno M. L. López-Casado M. Á (2019). Celiac immunogenic potential of α-gliadin epitope variants from *Triticum* and *Aegilops* species. *Nutrients* 22:E220. 10.3390/nu11020220 30678169 PMC6413208

[B55] Sánchez-León S. Gil-Humanes J. Ozuna C. V. Giménez M. J. Sousa C. Voytas D. F. (2018). Low-gluten, nontransgenic wheat engineered with CRISPR/Cas9. *Plant Biotechnol. J.* 16 902–910. 10.1111/pbi.12837 28921815 PMC5867031

[B56] Sapone A. Lammers K. M. Casolaro V. Cammarota M. Giuliano M. T. De Rosa M. (2011). Divergence of gut permeability and mucosal immune gene expression in two gluten-associated conditions: celiac disease and gluten sensitivity. *BMC Med.* 9:23. 10.1186/1741-7015-9-23 21392369 PMC3065425

[B57] Scherf K. A. Wieser H. Koehler P. (2018). Novel approaches for enzymatic gluten degradation to create high-quality gluten-free products. *Food Res. Int.* 110 62–72. 10.1016/j.foodres.2016.11.021 30029707

[B58] Schuppan D. (2000). Current concepts of celiac disease pathogenesis. *Gastroenterology* 119 234–242. 10.1053/gast.2000.8521 10889174

[B59] Schuppan D. Junker Y. Barisani D. (2009). Celiac disease: from pathogenesis to novel therapies. *Gastroenterology* 137 1912–1933. 10.1053/j.gastro.2009.09.008 19766641

[B60] Shewry P. R. Halford N. G. (2002). Cereal seed storage proteins: structures, properties and role in grain utilization. *J. Exp. Bot.* 53 947–958. 10.1093/jexbot/53.370.947 11912237

[B61] Shewry P. R. Napier J. A. Tatham A. S. (1995). Seed storage proteins: structures and biosynthesis. *Plant Cell* 7 945–956. 10.1105/tpc.7.7.945 7640527 PMC160892

[B62] Shi C. Xu L. L. (2009). Characters of cysteine endopeptidases in wheat endosperm during seed germination and subsequent seedling growth. *J. Integr. Plant Biol.* 51 52–57. 10.1111/j.1744-7909.2008.00778.x 19166494

[B63] Shindo T. van der Hoorn R. A. L. (2008). Papain-like cysprot: key players at molecular battlefields employed by both plants and their invaders. *Mol. Plant Pathol.* 1 119–125. 10.1111/j.1364-3703.2007.00439.x 18705889 PMC6640327

[B64] Sreenivasulu N. Usadel B. Winter A. Radchuk V. Scholz U. Stein N. (2008). Barley grain maturation and germination: metabolic pathway and regulatory network commonalities and differences highlighted by new mapman/pageman profiling tools. *Plant Physiol.* 146 1738–1758. 10.1104/pp.107.111781 18281415 PMC2287347

[B65] Stenman S. M. Lindfors K. Venäläinen J. I. Hautala A. Männistö P. T. Garcia-Horsman J. A. (2010). Degradation of coeliac disease-inducing rye secalin by germinating cereal enzymes: diminishing toxic effects in intestinal epithelial cells. *Clini. Exper. Immunol.* 161 242–249. 10.1111/j.1365-2249.2010.04119.x 20560983 PMC2909406

[B66] Stenman S. M. Venäläinen J. I. Lindfors K. Auriola S. Mauriala T. Kaukovirta-Norja A. (2009). Enzymatic detoxification of gluten by germinating wheat proteases: implications for new treatment of celiac disease. *Ann. Med.* 41 390–400. 10.1080/07853890902878138 19353359

[B67] Szewinska J. Siminska J. Bielawski W. (2016). The roles of cysteine proteases and phytocystatins in development and germination of cereal seeds. *J. Plant Physiol.* 207 10–21. 10.1016/j.jplph.2016.09.008 27771502

[B68] Tan-Wilson A. L. Wilson K. A. (2011). Mobilization of seed protein reserves. *Physiol. Plant.* 145 140–153. 10.1111/j.1399-3054.2011.01535.x 22017287

[B69] Tornkvist A. Liu C. Moschou P. N. (2019). Proteolysis and nitrogen: emerging insights. *J. Exp. Bot.* 70 2009–2019. 10.1093/jxb/erz024 30715465

[B70] Tye-Din J. A. Stewart J. A. Dromey J. A. Beissbarth T. van Heel D. A. Tatham A. (2010). Comprehensive, quantitative mapping of T cell epitopes in gluten in celiac disease. *Sci. Transl. Med.* 2 1–14. 10.1126/scitranslmed.3001012 20650871

[B71] van der Hoorn R. A. (2008). Plant proteases: from phenotypes to molecular mechanisms. *Annu. Rev. Plant Biol.* 59 191–223. 10.1146/annurev.arplant.59.032607.092835 18257708

[B72] van der Hoorn R. A. L. Jones J. D. G. (2004). The plant proteolytic machinery and its role in defense. *Curr. Opin. Plant Biol.* 7 400–407. 10.1016/j.pbi.2004.04.003 15231262

[B73] Velasco-Arroyo B. Diaz-Mendoza M. Santamaria M. E. Gonzalez-Melendi P. Gomez-Sanchez A. Arnaiz A. (2016). Senescence-associated genes in response to abiotic/biotic stresses. *Prog. Bot.* 79 89–109. 10.1007/124-2017-1

[B74] Velasco-Arroyo B. Martínez M. Díaz I. Diaz-Mendoza M. (2018). Differential response of silencing HvIcy2 barley plants against *Magnaporthe oryzae* infection and light deprivation. *BMC Plant Biol.* 18:337. 10.1186/s12870-018-1560-6 30522452 PMC6282322

[B75] Washio K. Ishikawa K. (1994). Organ-specific and hormone-dependent expression of genes for serine carboxypeptidases during development and following germination of rice grains. *Plant Physiol.* 105 1275–1280. 10.1104/pp.105.4.1275 7972496 PMC159459

[B76] Zhang N. Jones B. L. (1995). Characterization of germinated barley endoproteolytic enzymes by two dimensional gel electrophoresis. *J. Cereal Sci.* 21 145–153. 10.1016/0733-5210(95)90030-6

