# Supplementary material for: Plant Proteases: From Key Enzymes in Germination to Allies for Fighting Human Gluten-Related Disorders
Source: Front Plant Sci. 2019 May 29;10:721. doi: 10.3389/fpls.2019.00721 (PMC6548828; doi:10.3389/fpls.2019.00721)
Supplement: Supplementary file 1 [file Table_1.docx]

**MATERIALS AND METHODS**

*Gliadin purification from wheat*

Gliadins from six different wheat genotypes (‘Perico’, THA1, THA7, THA53, BW2003 and BW208) were extracted from flour as described in **Gil-Humanes et al. (2012**) and freeze dried.

*Expression, purification and activation of recombinant cysteine proteases from E.coli*

cDNA fragments covering the whole HvPap-1, -4, -6 and -16 open reading frames (ORFs), apart from their signal peptide sequences, were amplified by PCR and inserted into the expression vector pRSETB (Invitrogen). The recombinant plasmids were introduced in *E.coli* strain BL21 (**Martínez et al., 2009**). Proteases with C- terminal His6 tags were purified with Ni-NTA agarose columns (Qiagen). The purification process was checked by SDS-PAGE. Final protein concentrations were quantified by Nano-Drop (**Martínez et al., 2009**). Activation of CysProt by pepsin, when necessary, was carried out as described in **Cambra et al. (2012**). Protease activity of HvPap-1, -4, -6 and -16 was determined using the fluorogenic substrate Z-FR-AMC (N-carbobenzoxyloxy-Phe-Arg-7-amido-4-methylcoumarin) according to **Martínez et al. (2009**).

*Gliadin-proteases treatments*

Eight μg of gliadins from Perico, THA1, THA7, THA53, BW2003 and BW208 cultivars, were incubated for 1 h and 12 h at 28 ºC with 2 μg of each CysProt, HvPap-1, -4, -6 and -16 in 100 mM sodium phosphate pH 6.0 buffer (10 mM cysteine, 1 mM EDTA and 0.01% Brij35). This ratio of enzyme vs substrate (1:4) is efficient for a gliadin-degrading enzyme and has been optimized according to enzymatic assays tuned by our group (**Martínez et al., 2009, Cambra et al., 2012**). Stability of gliadins at 1 h and 12 h without CysProt was also proven, as well as a control treatment containing 6 μg of pepsin, the same concentration used to activate cysteine-proteases (**Cambra et al., 2012**), to check degradation due to pepsin in the sample. Cold acetone was added to gliadin-protease treatments in a ratio of 5:1(v/v) to stop the reaction and precipitate the proteins. Samples were stored for at least 3 h at -20 ºC before centrifugation for 10 min at 13.000xg. Pellets were dried and resuspended in loading buffer (12.5 mM Tris-HCl pH 6.8, 0.25% SDS, 2.5% β-mercaptoethanol, 0.01% bromophenol blue and 3.75% glycerol). After this process, samples were loaded in SDS-PAGE gels or stored at -20 ºC.

*Western blot analysis*

After protein separation on a SDS-PAGE electrophoresis, gel was electrotransferred onto a nitrocellulose membrane (GE Healthcare). Membrane was rinsed in Phosphate Buffer Saline (PBS: 137 mM sodium chloride, 2.7 mM potassium chloride, 10 mM sodium phosphate dibasic, 2 mM potassium phosphate monobasic, 0.1% Tween 20) and incubated for 2 h in a blocking buffer containing PBS and 5% (w/v) skim milk. After rinsing the membrane three times in PBS with 1, 0.1 and 0.01% of Tween 20, respectively, immunoblotting was performed with Gluten Tox G12 HRP-conjugate antibody, 200 X (Biomedal diagnostics). The G12 antibody was raised against the highly immunotoxic 33-mer peptide of the α- gliadin protein that induces celiac disease (**Morón et al., 2008a**). More specifically, the region of recognition within the 33-mer is the hexameric epitope QPQLPY. This recognition sequence is repeated three times within the gliadin 33-mer peptide. The G12 antibody recognizes immunotoxic prolamins from wheat, barley, rye and also from some varieties of oat (**Morón et al., 2008b**). Optimum dilution of the antibody at 1:4000 in PBS was used. After 2 h of incubation with the antibody, membranes were washed with PBS and then reacted with Super Signal West Pico (Thermo Scientific) for detection with RP2 PLUS Medical X-Ray Film (Agfa HealthCare).

**References:**

Cambra, I., Martínez, M., Dáder, B., González-Melendi, P., Gandullo, J., Santamaría, M.E., Diaz, I. (2012). A cathepsin F-like peptidase involved in barley grain protein mobilization, HvPap-1, is modulated by its own propeptide and by cystatins. *J. Exp. Bot.* 63, 4615-4629. doi: 10.1093/jxb/ers137.

Gil-Humanes, J., Pistón, F., Giménez, M.J., Martín, A., Barro, F. (2012). The intogression of RNAi silencing of γ-gliadins into commercial lines of bread wheat changes the mixing and technological properties of the dough. *PLoS ONE*, 7, e45937. doi: 10.1371/journal.pone.0045937.

Martinez, M., Cambra, I., Carrillo, L., Diaz-Mendoza, M., Diaz, I. (2009). Characterization of the entire cystatin gene family in barley and their target cathepsin L-like cysteine proteases, partners in the hordein mobilization during seed germination. *Plant Physiol.* 151, 1531-1545. doi: 10.1104/pp.109.146019.

Morón, B., Cebolla, A., Manyani, H., Alvarez-Maqueda, M., Megías, M., Thomas, M.C., López, M.C., Sousa, C. (2008a). Sensitive detection of cereal fractions that are toxic to celiac disease patients by using monoclonal antibodies to a main immunogenic wheat peptide. *Food Nutr. Res*. 87, 405-14. doi: https://doi.org/10.1093/ajcn/87.2.405

Morón, B., Bethune, M.T., Comino, I., Manyani, H., Ferragud, M., López, M.C., Cebolla, A., Khosla, C., Sousa, C. (2008b). Toward the assessment of food toxicity for celiac patients: characterization of monoclonal antibodies to a main immunogenic gluten peptide. *PLoS One*. 3, e2294. doi: https://doi.org/10.1371.
